# Nut Consumption Is Associated with Lower Risk of Metabolic Syndrome and Its Components in Type 1 Diabetes

**DOI:** 10.3390/nu13113909

**Published:** 2021-10-30

**Authors:** Aila J. Ahola, Carol M. Forsblom, Valma Harjutsalo, Per-Henrik Groop

**Affiliations:** 1 Folkhälsan Institute of Genetics, Folkhälsan Research Center, 00290 Helsinki, Finland; aila.ahola@helsinki.fi (A.J.A.); carol.forsblom@hus.fi (C.M.F.); valma.harjutsalo@helsinki.fi (V.H.); 2 Department of Nephrology, University of Helsinki and Helsinki University Hospital, 00290 Helsinki, Finland; 3 Research Program for Clinical and Molecular Metabolism, Faculty of Medicine, University of Helsinki, 00014 Helsinki, Finland; 4 National Institute for Health and Welfare, 00300 Helsinki, Finland; 5 Department of Diabetes, Central Clinical School, Monash University, Melbourne, VIC 3004, Australia

**Keywords:** nut consumption, metabolic syndrome, type 1 diabetes

## Abstract

Although nut consumption has been associated with several health benefits, it has not been investigated in individuals with type 1 diabetes. Therefore, our aim was to assess nut consumption and its association with metabolic syndrome in adult individuals with type 1 diabetes taking part in the Finnish Diabetic Nephropathy Study. The nut intake of the 1058 participants was assessed from 3-day food records that were completed twice, and the number of weekly servings, assuming a serving size of 28.4 g, was calculated. Metabolic syndrome was defined as the presence of ≥3 of the cardiovascular risk factors: central obesity, high blood pressure (≥130/85 mmHg or use of antihypertensive medication), high triglyceride concentration (≥1.70 mmol/L or use of lipid-lowering medication), low HDL-cholesterol concentration (<1.00 mmol/L in men and <1.30 mmol/L in women or use of lipid-lowering medication), and hyperglycaemia. Overweight/obesity was defined as a BMI ≥25 kg/m^2^. HbA_1c_ > 59 mmol/mol (>7.5%) was used as a criterion for suboptimal glycaemic control. Of the 1058 (mean age 46 years, 41.6% men) participants, 689 (54.1%) reported no nut intake. In the remaining sample, the median weekly nut intake was 40.8 g. In the adjusted models, higher nut intake, as the continuous number of weekly servings and the comparison of those with <2 and ≥2 weekly servings, was associated with lower metabolic syndrome score, waist circumference, HbA_1c_, and BMI. Nut consumption as a continuous variable was negatively associated with the presence of metabolic syndrome, its blood pressure, triglyceride, and HDL-cholesterol components, and suboptimal glycaemic control. Consumption of ≥2 weekly servings was associated with lower odds of suboptimal glycaemic control (by 51.5%), overweight/obesity (by 33.4%), and metabolic syndrome (by 51.8%) and meeting the waist (by 37.3%), blood pressure (by 44.5%), triglyceride (by 37.7%), and HDL-cholesterol (by 36.2%) components of the metabolic syndrome. In conclusion, a weekly nut intake of ≥2 servings was beneficially associated with all the components of the metabolic syndrome in type 1 diabetes. The causality of this association will need to be investigated.

## 1. Introduction

Nuts, as part of a healthy diet, have gained increasing attention as they are good sources of unsaturated fatty acids, fiber, vegetable protein, minerals, and bioactive compounds [1]. Given that nuts are low in carbohydrates, they minimally contribute to postprandial glycaemia, which is important to individuals with diabetes. However, beyond affecting postprandial glycaemia, nuts also have other beneficial health effects. Amongst others, nut consumption has been associated with a lower risk of coronary arterial disease and related mortality [2,3,4]. Based on the cumulated evidence, the U.S. Food and Drug Administration issued a qualified health claim in 2003 stating that the daily consumption of 1.5 ounces (42.6 g) of most nuts, as part of a healthy diet, may reduce the risk of heart disease [5].

Metabolic syndrome, a cluster of cardiovascular risk factors, is an important cause of morbidity and mortality worldwide [6]. The components of the metabolic syndrome, including hyperglycaemia, high blood pressure, high triglyceride concentrations, low HDL-cholesterol concentrations, and central obesity [7], are also frequently observed in individuals with type 1 diabetes [8]. There is also evidence that metabolic syndrome in type 1 diabetes is associated with an increased risk of long-term vascular complications [9]. Although individuals with type 1 diabetes are at a high risk of adverse vascular events, the potential health effects of nut consumption have not been investigated in this vulnerable patient group. Instead, the association between nut consumption and metabolic syndrome has been investigated in a number of other populations. In the Prevención con Dieta Mediterránea (PREDIMED) trial, in individuals with a high risk of cardiovascular diseases, the consumption of more than three weekly servings of nuts was associated with lower odds of the metabolic syndrome compared to those reporting less than one weekly serving [10]. In the SUN Project with Spanish university graduates, those reporting the consumption of ≥2 weekly servings had a significantly lower risk of future metabolic syndrome compared to the non-consumers [11]. Our aim was, therefore, to assess nut consumption in a well-defined sample of Finnish adults with type 1 diabetes and to investigate the association between nut intake and the presence of metabolic syndrome and its components.

## 2. Materials and Methods

Data for the current analyses were collected from The Finnish Diabetic Nephropathy (FinnDiane) Study, which is an ongoing nation-wide multicenter study for identifying the risk factors for diabetes complications in type 1 diabetes. Since the launch of the FinnDiane Study in 1997, more than 5000 individuals have been recruited at 92 sites, including 5 university hospitals, 16 central hospitals, most of the regional hospitals, and several primary healthcare centers around Finland (see the electronic supplementary material for a list of the centers). The nutrition sub-study of the FinnDiane Study was initiated in 2007; thus, the number of participants in the current study (*n* = 1058) is lower. In this study, type 1 diabetes was defined as the onset of diabetes prior to the age of 40 years with permanent insulin treatment initiated within a year from the diabetes diagnosis. For these analyses, we included data from all adult (age ≥18 years) participants reporting plausible energy intake (3350–16,740 kJ/d or 800–4000 kcal/d). Individuals with an estimated glomerular filtration rate (eGFR) <30 mL/min/1.73 m^2^, undergoing dialysis, or having a renal transplant were excluded. The Ethics Committee of Helsinki and Uusimaa Hospital District approved the study protocol (Ethics committee reference number 491/E5/06). The study was carried out in accordance with the Declaration of Helsinki and its later amendments. Written informed consent was obtained from all individuals prior to study participation.

### 2.1. Study Visit

At the FinnDiane Study visit, participants were thoroughly investigated, as previously explained in detail [12]. Being of importance to the current analyses, waist circumference, weight, and height were measured. Following a 10-min rest, seated blood pressure was measured twice. The mean of the measurements was used in the analyses. Blood was drawn for the subsequent central analyses of serum lipid, lipoprotein, and creatinine concentrations, performed at the Helsinki University Hospital laboratory. Glycated haemoglobin (HbA_1c_) was measured locally using a standardized assay. Serum creatinine concentration was used to calculate the eGFR [13]. Current smoking, insulin dosing, and medication use were self-reported on questionnaires.

### 2.2. Outcome Variables 

Metabolic syndrome was defined according to the criteria by Alberti et al. [7]. Accordingly, metabolic syndrome was assumed with the presence of 3 or more of the following criteria: waist circumference ≥94 cm in men and ≥80 cm in women, triglyceride concentration ≥1.70 mmol/L (or use of lipid-lowering medication), HDL-cholesterol concentration <1.00 mmol/L in men and <1.30 mmol/L in women (or use of lipid-lowering medication), blood pressure ≥130/85 mmHg (or use of antihypertensive medication), and fasting glucose concentration ≥6.11 mmol/L (or diabetes diagnosis). As all the participants had type 1 diabetes, the total metabolic syndrome score ranged between 1 and 5. Body mass index (BMI) was treated as a continuous variable, and we additionally divided the participants into those with normal weight (BMI < 25 kg/m^2^) and those with overweight or obesity (BMI ≥ 25 kg/m^2^). Glycaemic control, measured as HbA_1c_, was also used both as a continuous variable and as a dichotomous variable. When dichotomizing, suboptimal glycaemic control was defined as HbA_1c_ > 59 mmol/mol or >7.5%.

### 2.3. Dietary Intake

In the FinnDiane Study, two separate methods were used to assess dietary intake, as previously described [14]. First, the participants filled out a validated [15] diet questionnaire in which the consumption of the most common food items, in Finland, was reported. Thereafter, the participants twice completed a three-day food record with a 2–3 month interval. The consecutive days for this recording were allocated and comprised of two weekdays and one weekend day. In the record, all the foods and drinks consumed were reported using household measures or information on the food labels to estimate the portion sizes. The continuation of habitual practices during the recording days was emphasized. In the same record, participants also reported their physical activity (type of activity, its strenuousness, and its duration). For the purpose of the current study, the diet questionnaire was used for calculating a diet score, as previously explained in detail [16]. Briefly, the entries in the questionnaire were compared against the Finnish dietary recommendations, and their closer adherence yielded a higher score. AivoDiet software (version 2.0.2.3, AIVO, Turku, Finland) was used to calculate the total energy intake from the data collected with the food records. Data on nut consumption were retrieved from the food records. Here, data on the reported amounts of almonds, Brazil nuts, cashew nuts, hazel nuts, macadamia nuts, pecans, pine nuts, pistachio nuts, walnuts, and, although botanically a legume, peanuts were collected. Based on the food record entries, the weekly amount was calculated for each nut type. For example, if over the six recording days a consumption of 50 g of cashew nuts was reported, the weekly dose was calculated as (50 g/6 days) × 7 days. By adding up the weekly doses of all the individual nuts, we obtained a total weekly nut intake. Using the serving size of 28.4 g (1 oz), we further calculated a weekly number of servings for each participant. Finally, using the physical activity data reported in the records, the total amount of physical activity as the metabolic equivalent of task (MET) minutes was calculated by multiplying the duration of the activity by the activity- and intensity-specific metabolic equivalent.

### 2.4. Statistical Analyses

Data are presented as frequencies for categorical variables, medians (interquartile ranges) for continuous variables with skewed distribution (normality was assessed with the Kolmogorov–Smirnov test), and means ± standard deviations for continuous variables with normal distribution. In these respective variables, between-group comparisons were conducted with the Chi-squared test, Mann–Whitney U-test, and independent samples’ *t*-test. Data on nut consumption were treated as continuous variables (grams per week and servings per week). In addition, the sample was divided into those weekly consuming <2 and ≥2 servings of nuts. Differences in the background variables (sex, age, smoking status, diet score, total energy intake, physical activity, insulin dosing, and eGFR) between these two groups were investigated, and variables with sufficient between-group difference (*p* < 0.1) were chosen as confounders for the multivariable models. For sensitivity analyses, we also compared those reporting the consumption of <1 and ≥1 weekly servings (results shown in the supplementary material). Generalized linear regression was used to study the associations between nut consumption and the continuous outcome variables (metabolic syndrome score, waist circumference, systolic and diastolic blood pressures, triglyceride and HDL-cholesterol concentrations, HbA_1c_, and BMI). Logistic regression analysis was used to study the associations between nut consumption and the categorical outcome variables (metabolic syndrome, the presence of the individual components of the metabolic syndrome, suboptimal glycaemic control, and the presence of overweight or obesity). Analyses were conducted with IBM SPSS Statistics for Windows, Version 25.0 (IBM, Armonk, NY, USA). A two-tailed *p*-value < 0.05 was chosen to designate statistical significance.

## 3. Results

Dietary data were available from 1058 individuals (mean ± standard deviation age 46 ± 14 years, 41.6% men). Since the initiation of the dietary data collection, 1867 individuals had been investigated at the FinnDiane Study visit by the time of data retrieval. Thus, the percentage of individuals completing the food record was 56.7%. Individuals not completing the food records were more frequently men, were younger, and were more frequently current smokers (Supplementary Table S1). In addition, the non-responders had a worse health profile as they more frequently had suboptimal glycaemic control and metabolic syndrome. In addition, they had higher diastolic blood pressure and triglyceride concentration but lower HDL-cholesterol concentration. Instead, the two groups were comparable in their median systolic blood pressure, total cholesterol concentration, and BMI. 

In all, 689 (65.1%) of the sample reported no nut intake. In the remaining sample, the reported median (interquartile range) weekly total nut dose was 40.8 (11.7–115.6) g. Among those reporting nut intake, peanuts were consumed in the highest quantities (27.1 g/week), followed by cashew nuts (26.1 g/week), almonds (13.0 g/week), walnuts (7.2 g/week), hazel nuts (6.8 g/week), pecans (2.9 g/week), macadamia nuts (0.8 g/week), pistachio nuts (0.7 g/week), Brazil nuts (0.5 g/week), and pine nuts (0.2 g/week).

Those consuming at least two weekly servings (*n* = 156, 14.7%), were less frequently men, were more physically active, and reported higher total energy intake and lower total insulin dose per body weight compared to those with no or low nut intake (Table 1). Instead, the two groups were comparable with respect to smoking, diet score, and eGFR. Albeit not significantly, those with higher nut intake were younger. The metabolic syndrome and overweight/obesity were more frequently observed in the group with no or low nut intake, while the group reporting the consumption of ≥2 weekly servings of nuts had better glycaemic control and lower systolic blood pressure, triglyceride concentration, waist circumference, and BMI.

In the generalized linear regression analyses, adjusted for age, sex, total energy intake, insulin dose, and physical activity, the continuous nut intake was negatively associated with the metabolic syndrome score, waist circumference, HbA_1c_, and BMI (Table 2). In addition, the consumption of ≥2 weekly servings of nuts was associated with lower adjusted metabolic syndrome score, waist circumference, HbA_1c_, and BMI, as compared to those with no or low intakes. These observations were repeated when comparing those reporting <1 and ≥1 weekly servings (Supplementary Table S2).

In the multivariable logistic regression analyses, nut consumption, measured as a continuous variable, was negatively associated with the presence of metabolic syndrome, and its blood pressure, triglyceride, and HDL-cholesterol components, and suboptimal glycaemic control (Table 3). Compared to those with no or low nut consumption, individuals consuming ≥2 weekly servings of nuts had 51.5% lower odds of suboptimal glycaemic control, 33.4% lower odds of overweight/obesity, and 51.8% lower odds of having metabolic syndrome. In addition, the odds of meeting the waist (by 37.3%), blood pressure (by 44.5%), triglyceride (by 37.7%), and HDL-cholesterol (by 36.2%) components of the metabolic syndrome were significantly lower in those with higher nut intake. When comparing individuals reporting the consumption of <1 and ≥1 weekly servings of nuts, the odds of metabolic syndrome, the blood pressure component, suboptimal glycaemic control, and overweight/obesity were lower in those with higher intake (Supplementary Table S3).

## 4. Discussion

In this study, the consumption of nuts equaling or exceeding two weekly doses (i.e., ≥56.8 g) was associated with reduced odds of meeting the criteria for the metabolic syndrome and all its individual components in adult individuals with type 1 diabetes. In addition, compared to those with no or low nut intake, the odds of good glycaemic control and normal body weight were significantly higher in those reporting the consumption of at least two weekly doses. Notably, lower doses were also beneficial as the consumption of at least one weekly serving was associated with lower odds of metabolic syndrome, the blood pressure component, suboptimal glycaemic control, and overweight or obesity. 

To the best of our knowledge, the association between nut intake and metabolic syndrome in individuals with type 1 diabetes has not been previously investigated. However, studies in other populations are available. For example, in the cross-sectional analyses of the Prevención con Dieta Mediterránea (PREDIMED) trial, among individuals with high cardiovascular disease risk, compared to those reporting the consumption of less than one serving of nuts per week, those with over three weekly servings had lower odds of the metabolic syndrome [10]. Following the randomization of the PREDIMED study participants into a Mediterranean diet supplemented with virgin olive oil, a Mediterranean diet supplemented with mixed nuts, or a low-fat diet, those in the group supplementing with nuts had a significantly reduced prevalence of the metabolic syndrome after one year [17]. In the prospective SUN Project of 9887 university graduates initially free of metabolic syndrome, individuals consuming ≥2 servings of nuts per week presented a 32% lower risk of developing the metabolic syndrome after 6 years of follow-up compared to those who never or almost never consumed nuts [11].

Independent of factors such as total energy intake and physical activity, we observed lower BMI, lower odds of overweight/obesity, lower waist circumference, and lower odds of meeting the waist component of the metabolic syndrome in those with higher nut consumption. Moreover, in the cross-sectional analysis of the PREDIMED study, higher nut consumption was associated with lower odds of obesity and lower odds of fulfilling the criteria for abdominal obesity [10], and after one year into the study, the prevalence of the waist component of the metabolic syndrome was reduced in the supplemental nut group as compared to the low-fat group [17]. In the National Health and Nutrition Examination Survey (NHANES), compared to non-consumption, nut consumption was associated with a 12% lower risk of overweight or obesity [18]. In the Nurses’ Health Study, women who reported eating nuts ≥2 times per week had lower mean weight gain and lower risk of obesity over the 8-year follow-up, compared to those who rarely ate nuts [19]. However, two meta-analyses of intervention studies concluded that nut consumption had no impact on waist circumference [20] or body weight [21].

In the current study, higher nut consumption was not associated with the continuous measures of systolic or diastolic blood pressure but was associated with lower odds of meeting the blood pressure component of the metabolic syndrome. Contrary to our observations, no association between nut consumption and the blood pressure component was observed in the cross-sectional analyses of the PREDIMED data [10]. However, at a one-year follow-up, individuals supplementing with nuts exhibited a reduced prevalence of high blood pressure relative to the low-fat group [17]. In the NHANES, nut consumption, as compared to non-consumption, was associated with a 13% lower risk of hypertension [18]. Among participants in the Physicians’ Health Study, free from hypertension at the baseline and relative to individuals reporting no nut consumption, those reporting consuming nuts ≥7 times per week had reduced odds of incident hypertension [22]. However, after adjusting for BMI, an inverse relation between nut intake and hypertension was only evident in lean subjects. Similarly, in the SUN prospective cohort, followed up for a median of 4.3 years, self-reported nut intake was not associated with incident hypertension [23]. While a number of meta-analyses of controlled interventions have reported no effect of nut intake on blood pressure [20,24,25], in their meta-analysis Mohammadifard et al. concluded that nut consumption led to a significant reduction in systolic blood pressure only in individuals without type 2 diabetes, but not in the total population [26].

The odds of meeting both the triglyceride and HDL-cholesterol components of the metabolic syndrome in this study were significantly lower in those reporting higher nut intakes. However, nut intake was not associated with the continuous measurements of these lipid variables. While no cross-sectional associations were observed between nut intake and dyslipidaemia in the PREDIMED study [10], the prevalence of elevated triglyceride concentration was reduced in the nut-supplemented individuals one year after the commencement of the intervention [17]. Compared to the non-consumers in the NHANES, those reporting nut consumption had a 20% lower risk of low HDL-cholesterol concentration [18]. Instead, in the Nurses’ Health Study, nut consumption was not related to HDL-cholesterol concentration, but higher intakes were associated with lower LDL-cholesterol, non-HDL-cholesterol, total cholesterol, and apolipoprotein B-100 concentrations [27]. In a meta-analysis of randomized controlled trials of tree-nut interventions, reduction in triglyceride concentration was observed with no changes evident in the HDL-cholesterol concentration [20]. Finally, in another meta-analysis including trials with walnut-enriched diets, reductions in total cholesterol, LDL-cholesterol, and triglyceride concentrations, as compared to a control diet, were reported [25].

Of specific importance to individuals with type 1 diabetes, we observed better glycaemic control with increasing nut intake. While a number of previous trials have shown reduced post-prandial blood glucose concentrations related to nut consumption, suggestive of at least a short-term beneficial glycaemic effect [28,29,30], the effects on other measures of glucose control are mixed. For example, in the cross-sectional analyses of the PREDIMED data, nut consumption was not associated with the fasting blood glucose concentration but compared to individuals consuming less than one weekly serving of nuts, those with over three weekly servings had lower odds of diabetes [10]. Moreover, in a meta-analysis of 12 randomized controlled trials including participants with type 2 diabetes, a median daily consumption of 56 g of tree nuts for a median duration of 8 weeks significantly reduced HbA_1c_ and fasting glucose concentration compared to the control diets but had no effects on fasting insulin and HOMA-IR [31]. Instead, in another meta-analysis of randomized controlled trials with a 3-month median intervention duration, the consumption of tree nuts and peanuts was beneficially associated with HOMA-IR and fasting insulin concentration but had no effect on fasting blood glucose concentration or HbA_1c_ [32]. Yet, in another meta-analysis of randomized controlled trials, walnut consumption had no impact on the markers of blood glucose control, including fasting glucose, HbA_1c_, fasting insulin, and HOMA-IR [33].

Several mechanisms may explain the health benefits related to nut consumption. For example, in a meta-analysis of 23 randomized controlled trials, long-term (≥12 week) nut intake significantly reduced the concentrations of the intercellular adhesion molecule and the vascular cell adhesion molecule [34], particles involved in inflammatory processes. Various phytosterols found in nuts likely interfere with cholesterol absorption and thus have the potential to bring about cholesterol-lowering effects [35]. Moreover, especially when replacing saturated fatty acids, the high unsaturated fatty acid content of nuts has the potential to beneficially impact the lipid profile [36]. Phenolic acids attenuate oxidative stress, leading to blood pressure lowering through improved endothelial function and nitric oxide bioavailability in the arterial vasculature [37]. Important for weight control, nut consumption has been shown to reduce glycaemic response and increase satiety [38]. Finally, nut consumption has also been associated with an increased relative abundance of the genera *Clostridium*, *Lachnospira*, and *Roseburia* [39] in the gut, all of which are known butyrate producers [40]. Butyrate, on the other hand, is an important source of energy for intestinal colonocytes and plays a role in the regulation of cell proliferation and differentiation [41].

A large and well-defined sample of individuals with type 1 diabetes and the use of a food record, devoid of recall bias, to assess dietary intake are the major strengths of this study. However, the study is observational and does not allow the assessment of cause-effect relations. In addition, as is the case in epidemiological trials, some selection bias may have taken place as individuals who are more interested in their health are more likely to take part in health-related studies. Indeed, those not completing the food record had an overall worse health profile compared to those included in this study. The likely effect of this bias is the dilution of the current observations. It should also be noted that there are no universally accepted cut-off values for dichotomizing individuals into those with high and low nut intakes. The current decision to divide the sample into those reporting <2 or ≥2 weekly servings was based on the previous literature [11,19] and may be considered somewhat arbitrary. However, for sensitivity analyses, we additionally divided the sample into those reporting <1 or ≥1 weekly servings and observed that most of the original observations remained statistically significant. Finally, nut consumption may be associated with an overall healthy lifestyle, and while we considered several important confounders, the potential of residual confounding cannot be ruled out.

## 5. Conclusions

In conclusion, in individuals with type 1 diabetes this is the first time it has been shown that nut consumption is associated with reduced odds of the metabolic syndrome and all its individual components. While intervention trials are needed to establish the potential causal role of nuts for the health of individuals with type 1 diabetes, incorporating nuts in an overall healthy diet may well be already justified at this stage.

## Figures and Tables

**Table 1:** Participant characteristics divided by the amount of nut consumption.

	<2 Weekly Servings *n* = 902 (85.3%)	≥2 Weekly Servings *n* = 156 (14.7%)	*p*
Men %	42.9	34.0	0.043
Age, years	47 (36–57)	43 (34–55)	0.054
Current smoker, %	11.8	8.0	0.208
Diet score	11 (9–14)	11 (9–14)	0.811
Energy intake, MJ	7.6 (6.4–9.1)	8.4 (7.3–9.8)	<0.001
Physical activity, METmin/d	283 (154–465)	344 (210–593)	<0.001
Insulin dose/kg	0.58 (0.46–0.75)	0.51 (0.38–0.65)	<0.001
eGFR, ml/min/1.73 m^2^	100 (86–112)	101 (89–111)	0.392
Metabolic syndrome, %	67.1	44.9	<0.001
Metabolic syndrome score	3 (2–4)	2 (2–4)	<0.001
SBP, mmHg	135 (123–149)	131 (122–144)	0.008
DBP, mmHg	77 ± 9	76 ± 8	0.205
Total cholesterol, mmol/L	4.6 (4.0–5.2)	4.5 (3.9–5.1)	0.282
HDL-cholesterol, mmol/L	1.6 (1.3–1.9)	1.7 (1.4–1.9)	0.166
Triglycerides, mmol/L	0.95 (0.74–1.27)	0.77 (0.63–1.09)	<0.001
HbA_1c_, mmol/mol	64 (56–72)	58 (51–66)	<0.001
HbA_1c_, %	8.0 (7.3–8.7)	7.5 (6.8–8.2)	<0.001
Waist circumference, cm	88 (80–97)	83 (75–91)	<0.001
Overweight/obese, %	57.1	43.6	0.002
BMI, kg/m^2^	25.7 (23.3–28.6)	24.5 (22.9–26.7)	<0.001

Data are shown as frequency for categorical variables, median (interquartile range) for continuous variables with skewed distribution and mean ± standard deviation for continuous variables with normal distribution. Between-group comparisons were conducted with Chi-squared test, Mann–Whitney U-test, and independent samples’ *t*-test, respectively. One serving of nuts equals 28.4 g. METmin/d, metabolic equivalent of task minutes per day; eGFR, estimated glomerular filtration rate; SBP, systolic blood pressure; DBP, diastolic blood pressure; HbA_1c_, glycated haemoglobin; overweight/obese, body mass index ≥25 kg/m^2^; BMI, body mass index.

**Table 2:** Association between nut consumption and metabolic syndrome score, the continuous measures of the individual components of the metabolic syndrome, HbA_1c_, and body mass index.

	Servings per Week		<2 Weekly Servings	≥2 Weekly Servings	
	B (95% CI)	*p*	Mean (95% CI)	Mean (95% CI)	*p*
Metabolic syndrome score	−0.051 (−0.079–−0.022)	<0.001	3.2 (3.1–3.3)	2.8 (2.6–3.0)	<0.001
Waist circumference, cm	−0.286 (−0.562–−0.010)	0.042	89 (88–90)	86 (84–88)	0.005
SBP, mmHg	−0.343 (−0.712–0.027)	0.069	136 (135–137)	135 (132–137)	0.181
DBP, mmHg	−0.105 (−0.322–0.112)	0.342	77 (76–77)	76 (74–77)	0.462
Triglycerides, mmol/L	−0.004 (−0.020–0.012)	0.619	1.12 (1.07–1.16)	1.07 (0.96–1.18)	0.426
HDL-cholesterol, mmol/L	−0.004 (−0.013–0.006)	0.455	1.65 (1.62–1.68)	1.64 (1.58–1.71)	0.876
HbA_1c_, mmol/mol	−0.470 (−0.777–−0.162)	0.003	65 (64–66)	60 (58–62)	<0.001
BMI, kg/m^2^	−0.103 (−0.203–−0.004)	0.041	26.3 (26.0–26.6)	25.3 (24.6–26.0)	0.008

Generalized linear regression. Models are adjusted for age, sex, energy intake, insulin dosing, and physical activity. In addition, analyses with blood pressures as outcomes are adjusted for the use of antihypertensive medication, and analyses with lipid variables as outcomes are adjusted for the use of lipid-lowering medication. One serving of nuts equals 28.4 g. CI, confidence interval; SBP, systolic blood pressure; DBP, diastolic blood pressure; BMI, body mass index.

**Table 3:** Association between nut consumption and metabolic syndrome, its individual components, and overweight/obesity.

	Servings Per Week		<2 Weekly Servings	≥2 Weekly Servings	
	B (95% CI)	*p*		B (95% CI)	*p*
Metabolic syndrome	0.915 (0.866–0.968)	0.002	Ref.	0.482 (0.324–0.718)	<0.001
Waist component	0.957 (0.909–1.007)	0.090	Ref.	0.627 (0.434–0.905)	0.013
BP component	0.913 (0.861–0.967)	0.002	Ref.	0.555 (0.362–0.851)	0.007
Triglyceride component	0.925 (0.870–0.984)	0.013	Ref.	0.623 (0.407–0.952)	0.029
HDL-cholesterol component	0.940 (0.888–0.996)	0.036	Ref.	0.638 (0.424–0.958)	0.030
Suboptimal glycaemic control	0.936 (0.891–0.983)	0.008	Ref.	0.485 (0.340–0.693)	<0.001
Overweight/obesity	0.955 (0.909–1.002)	0.063	Ref.	0.666 (0.467–0.950)	0.025

Logistic regression analysis. Models are adjusted for age, sex, total energy intake, insulin dose, and physical activity. One serving of nuts equals 28.4 g. CI, confidence interval; Ref., reference; BP, blood pressure; suboptimal glycaemic control, HbA_1c_ > 59 mmol/mol (>7.5%); overweight/obesity, body mass index ≥ 25 kg/m^2^.

## Data Availability

According to the General Data Protection Regulation of the European Union (679/ 2016), the principles of data protection should apply to any information concerning an identified or identifiable natural person and that personal data which have undergone pseudonymization, which could be attributed to a natural person by the use of additional information should be considered to be information on an identifiable natural person. Thus, according to the GDPR, all pseudonymized data are considered personal data and cannot be published openly.

## Supplementary Materials

The following are available online at https://www.mdpi.com/article/10.3390/nu13113909/s1, Supplementary information of The Finnish Diabetic Nephropathy Study Centers. Table S1: Comparison of individuals in the FinnDiane Study completing and not completing the food record, Table S2: Association between nut consumption and metabolic syndrome score, the continuous measures of the individual components of the metabolic syndrome, HbA1c, and body mass index. Table S3: Association between nut consumption and metabolic syndrome, its individual components, and overweight/obesity.
