# Supplementary material for: Nut Consumption Is Associated with Lower Risk of Metabolic Syndrome and Its Components in Type 1 Diabetes
Source: Nutrients. 2021 Oct 30;13(11):3909. doi: 10.3390/nu13113909 (PMC8620387; doi:10.3390/nu13113909)
Supplement: Supplementary file 1 [file nutrients-13-03909-s001.zip › nutrients-1431102-supplementary.pdf]

Nut consumption is associated with lower risk of metabolic syndrome and its components in type 1 diabetes

Aila J. Ahola[1,2,3], Carol Forsblom[1,2,3], Valma Harjutsalo[1,2,3,4], Per-Henrik Groop[1,2,3,5]* on behalf of the FinnDiane Study Group

Supplementary information of The Finnish Diabetic Nephropathy Study Centers

Anjalankoski Health Center	S.Koivula, T.Uggeldahl
Central Finland Central Hospital, Jyväskylä	T.Forslund, A.Halonen, A.Koistinen, P.Koskiaho, M.Laukkanen, J.Saltevo, M.Tiihonen
Central Hospital of Åland Islands, Mariehamn	M.Forsen, H.Granlund, A.-C.Jonsson, B.Nyroos
Central Hospital of Kanta-Häme, Hämeenlinna	P.Kinnunen, A.Orvola, T.Salonen, A.Vähänen
Central Hospital of Kymenlaakso, Kotka	R.Paldanius, M.Riihelä, L.Ryysy
Central Hospital of Länsi-Pohja, Kemi	H.Laukkanen, P.Nyländen, A.Sademies
Central Ostrobothnian Hospital District, Kokkola	S.Anderson, B.Asplund, U.Byskata, P.Liedes, M.Kuusela, T.Virkkala
City of Espoo Health Center:	
Espoonlahti	A.Nikkola, E.Ritola
Tapiola	M.Niska, H.Saarinen
Samaria	E.Oukko-Ruponen, T.Virtanen
Viherlaakso	A.Lyytinen
City of Helsinki Health Center:	
Puistola	H.Kari, T.Simonen
Suutarila	A.Kaprio, J.Kärkkäinen, B.Rantaeskola
Töölö	P.Kääriäinen, J.Haaga, A-L.Pietiläinen
City of Hyvinkää Health Center	S.Klemetti, T.Nyandoto, E.Rontu, S.Satuli-Autere
City of Vantaa Health Center:	
Korso	R.Toivonen, H.Virtanen
Länsimäki	R.Ahonen, M.Ivaska-Suomela, A.Jauhiainen
Martinlaakso	M.Laine, T.Pellonpää, R.Puranen
Myyrmäki	A.Airas, J.Laakso, K.Rautavaara
Rekola	M.Erola, E.Jatkola
Tikkurila	R.Lönnblad, A.Malm, J.Mäkelä, E.Rautamo
Heinola Health Center	P.Hentunen, J.Lagerstam
Helsinki University Central Hospital, Department of Medicine, Division of Nephrology	M.Feodoroff, D.Gordin, O.Heikkilä, K.Hietala, J.Fagerudd, M.Korolainen, L.Kyllönen, J.Kytö, S.Lindh, K.Pettersson-Fernholm, M.Rosengård-Bärlund, A.Sandelin, L.Thorn, J.Tuomikangas, T.Vesisenaho, J.Wadén
Herttoniemi Hospital, Helsinki	V.Sipilä
Hospital of Lounais-Häme, Forssa	T.Kalliomäki, J.Koskelainen, R.Nikkanen, N.Savolainen, H.Sulonen, E.Valtonen
Hyvinkää Hospital	L. Norvio, A.Hämäläinen
Iisalmi Hospital	E.Toivanen
Jokilaakso Hospital, Jämsä	A.Parta, I.Pirttiniemi
Jorvi Hospital, Helsinki University Central Hospital	S.Aranko, S.Ervasti, R.Kauppinen-Mäkelin, A.Kuusisto, T.Leppälä, K.Nikkilä, L.Pekkonen
Jyväskylä Health Center, Kyllö	K.Nuorva, M.Tiihonen
Kainuu Central Hospital, Kajaani	S.Jokelainen, K.Kananen, M.Karjalainen, P.Kemppainen, A-M.Mankinen,
A.Reponen	
	M.Sankari
Kerava Health Center	H.Stuckey, P.Suominen
Kirkkonummi Health Center	A.Lappalainen, M.Liimatainen, J.Santaholma
Kivelä Hospital, Helsinki	A.Aimolahti, E.Huovinen
Koskela Hospital, Helsinki	V.Ilkka, M.Lehtimäki
Kotka Health Center	E.Pälikkö-Kontinen, A.Vanhanen
Kouvola Health Center	E.Koskinen, T.Siitonen
Kuopio University Hospital	E.Huttunen, R.Ikäheimo, P.Karhapää, P.Kekäläinen, M.Laakso, T.Lakka, E.Lampainen, L.Moilanen, S. Tanskanen L.Niskanen, U.Tuovinen, I.Vauhkonen, E.Voutilainen
Kuusamo Health Center	T.Kääriäinen, E.Isopoussu
Kuusankoski Hospital	E.Kilkki, I.Koskinen, L.Riihelä
Laakso Hospital, Helsinki	T.Meriläinen, P.Poukka, R.Savolainen, N.Uhlenius
Lahti City Hospital	A.Mäkelä, M.Tanner

Lapland Central Hospital, Rovaniemi	L.Hyvärinen, K.Lampela, S.Pöykkö, T.Rompasaari, S.Severinkangas, T.Tulokas
Lappeenranta Health Center	P. Erola, L.Härkönen, P.Linkola, T.Pekkanen, I.Pulli, E.Repo
Lohja Hospital	T.Granlund, K.Hietanen, M.Porrassalmi, M.Saari, T.Salonen, M.Tiikkainen,
Länsi-Uusimaa Hospital, Tammisaari	I.-M.Jousmaa, J.Rinne
Loimaa Health Center	A.Mäkelä, P.Eloranta
Malmi Hospital, Helsinki	H.Lanki, S.Moilanen, M.Tilly-Kiesi
Mikkeli Central Hospital	A.Gynther, R.Manninen, P.Nironen, M.Salminen, T.Vänttinen
Mänttä Regional Hospital	I.Pirttiniemi, A-M.Hänninen
North Karelian Hospital, Joensuu	U-M.Henttula, P.Kekäläinen, M.Pietarinen, A.Rissanen, M.Voutilainen
Nurmijärvi Health Center	A.Burgos, K.Urtamo
Oulaskangas Hospital, Oulainen	E.Jokelainen, P-L.Jylkkä, E.Kaarlela, J.Vuolaspuro
Oulu Health Center	L.Hiltunen, R.Häkkinen, S.Keinänen-Kiukaanniemi
Oulu University Hospital	R.Ikäheimo
Päijät-Häme Central Hospital	H.Haapamäki, A.Helanterä, S.Hämäläinen, V.Ilvesmäki, H.Miettinen
Palokka Health Center	P.Sopanen, L.Welling
Pieksämäki Hospital	V.Sevtsenko, M.Tamminen
Pietarsaari Hospital	M-L.Holmbäck, B.Isomaa, L.Sarelin
Pori City Hospital	P.Ahonen, P.Merisalo, E.Muurinen, K.Sävelä
Porvoo Hospital	M.Kallio, B.Rask, S.Rämö
Raahe Hospital	A.Holma, M.Honkala, A.Tuomivaara, R.Vainionpää
Rauma Hospital	K.Laine, K.Saarinen, T.Salminen
Riihimäki Hospital	P.Aalto, E.Immonen, L.Juurinen
Salo Hospital	A.Alanko, J.Lapinleimu, P.Rautio, M.Virtanen
Satakunta Central Hospital, Pori	M.Asola, M.Juhola, P.Kunelius, M.-L.Lahdenmäki, P.Pääkkönen, M.Rautavirta
Savonlinna Central Hospital	T.Pulli, P.Sallinen, M.Taskinen, E.Tolvanen, T.Tuominen H.Valtonen, A.Vartia, S-L.Viitanen
Seinäjoki Central Hospital	O.Antila, E.Korpi-Hyövälti, T.Latvala, E.Leijala, T.Leikkari, M.Punkari N.Rantamäki, H.Vähävuori
South Karelia Central Hospital, Lappeenranta	T.Ensala, E.Hussi, R.Härkönen, U.Nyholm, J.Toivanen
Tampere Health Center	A.Vaden, P.Alarotu, E.Kujansuu, H.Kirkkopelto-Jokinen, M.Helin, S.Gummerus, L.Calonius, T.Niskanen, T.Kaitala, T.Vatanen
Tampere University Hospital	P. Hannula, I.Ala-Houhala, R.Kannisto, T.Kuningas, P.Lampinen, M.Määttä, H.Oksala, T.Oksanen, A.Putila, H.Saha, K.Salonen, H.Tauriainen, S.Tulokas
Tiirismaa Health Center, Hollola	T.Kivelä, L.Petlin, L.Savolainen
Turku Health Center	A.Artukka, I.Hämäläinen, L.Lehtinen, E.Pyysalo, H.Virtamo, M.Viinikkala, M.Vähätalo
Turku University Central Hospital	K.Breitholz, R.Eskola, K.Metsärinne, U.Pietilä, P.Saarinen, R.Tuominen, S.Äyräpää
Vaajakoski Health Center	K.Mäkinen, P.Sopanen
Valkeakoski Regional Hospital	S.Ojanen, E.Valtonen, H.Ylönen, M.Rautiainen, T.Immonen
Vammala Regional Hospital	I.Isomäki, R.Kroneld, L.Mustaniemi, M.Tapiolinna-Mäkelä
Vasa Central Hospital	S.Bergkulla, U.Hautamäki, V-A.Myllyniemi, I.Rusk

Supplementary Table S1 Comparison of individuals in the FinnDiane Study completing and not completing the food record

	With food record<br>N=1058 (56.7%)	No food record<br>N=809 (43.3%)	p
Men, %	41.6	54.6	<0.001
Age, years	46.3 (35.8 – 56.7)	43.8 (33.9 – 52.5)	<0.001
Current smoker, %	11.2	18.6	<0.001
Suboptimal glycaemic control, %	62.8	72.9	<0.001
Metabolic syndrome, %	63.9	83.7	<0.001
Metabolic syndrome score	3 (2 – 4)	4 (3 – 5)	<0.001
SBP, mmHg	135 (123 – 148)	138 (126 – 149)	0.129
DBP, mmHg	77 (70 – 83)	79 (73 – 86)	0.015
Total cholesterol, mmol/l	4.54 (4.00 – 5.14)	4.53 (2.93 – 5.83)	0.502
HDL-cholesterol, mmol/l	1.59 (1.36 – 1.91)	1.45 (1.18 – 1.74)	<0.001
Triglycerides, mmol/l	0.93 (0.71 – 1.25)	1.07 (0.79 – 1.58)	<0.001
HbA_{1c}, mmol/mol	63 (55 – 72)	66 (58 – 76)	<0.001
HbA_{1c}, %	7.9 (7.2 – 8.7)	8.2 (7.5 – 9.1)	<0.001
BMI, kg/m^2	25.5 (23.2 – 28.4)	25.3 (22.7 – 28.4)	0.165
Overweight/obese, %	55.1	53.8	0.572

Data are shown as frequencies for categorical variables and median (interquartile range) for continuous variables. The between-group comparisons, in these respective variables, were conducted with Chi-squared test and Mann-Whitney U-test. Suboptimal glycaemic control, HbA1c >59 mmol/mol or >7.5%; SBP, systolic blood pressure; DBP, diastolic blood pressure; BMI, body mass index; overweight/obese, BMI ≥25 kg/m^2.

Supplementary Table S2 Association between nut consumption and metabolic syndrome score, the continuous measures of the individual components of the metabolic syndrome, HbA_{1c}, and body mass index

	<1 weekly servings	≥1 weekly servings	
	Mean (95% CI)	Mean (95% CI)	p
Metabolic syndrome score	3.2 (3.1 – 3.3)	3.0 (2.8 – 3.1)	0.016
Waist circumference, cm	89 (88 – 90)	87 (85 – 88)	0.027
SBP, mmHg	136 (135 – 137)	135 (133 – 137)	0.254
DBP, mmHg	77 (76 – 77)	76 (75 – 77)	0.463
Triglycerides, mmol/l	1.11 (1.07 – 1.16)	1.10 (1.01 – 1.19)	0.754
HDL-cholesterol, mmol/l	1.65 (1.62 – 1.67)	1.66 (1.61 – 1.72)	0.620
HbA_{1c}, mmol/mol	65 (64 – 66)	62 (60 – 63)	<0.001
BMI, kg/m^2	26.3 (26.0 – 26.6)	25.5 (25.0 – 26.1)	0.012

Generalized linear regression. Models are adjusted for age, sex, energy intake, insulin dosing, and physical activity. In addition, analyses with blood pressures as outcomes are adjusted for the use of antihypertensive medication and analyses with lipid variables as outcomes are adjusted for the use of lipid lowering medication. One serving of nuts equals to 28.4 grams. CI, confidence interval; SBP, systolic blood pressure; DBP, diastolic blood pressure; BMI, body mass index.

Supplementary Table S3 Association between nut consumption and metabolic syndrome, its individual components, and overweight/obesity

	<1 weekly servings	≥1 weekly servings	
		B (95% CI)	p
Metabolic syndrome	Ref.	0.617 (0.437 – 0.872)	0.006
Waist component	Ref.	0.752 (0.545 – 1.036)	0.081
BP component	Ref.	0.669 (0.460 – 0.973)	0.035
Triglyceride component	Ref.	0.778 (0.543 – 1.113)	0.169
HDL-cholesterol component	Ref.	0.777 (0.550 – 1.099)	0.153
Suboptimal glycaemic control	Ref.	0.568 (0.415 – 0.777)	<0.001
Overweight/obesity	Ref.	0.760 (0.536 – 0.995)	0.046

Logistic regression analysis. Models are adjusted for age, sex, total energy intake, insulin dose, and physical activity. One serving of nuts equals to 28.4 grams. CI, confidence interval; Ref, reference; BP, blood pressure; suboptimal glycaemic control, HbA_{1c} >59 mmol/mol (>7.5%); overweight/obesity, body mass index ≥25 kg/m^2.
